# Morpho-molecular assessment of nanomaterials in teleost fish wound healing: a systematic review

**DOI:** 10.1186/s12917-026-05934-6

**Published:** 2026-10-09

**Authors:** Abdelaziz Rabea, Iman Ibrahim, Hebatallah Mahgoub, Ahmed El-Shaieb

**Affiliations:** Pathology Department, Faculty of Veterinary Medicine, Mansoura University, Mansoura, 35516 Egypt

**Keywords:** Teleost fish, Wound healing, Nanomaterials, Systematic review, Histopathology, Aquaculture, Chitosan, Hyaluronic acid, *Oreochromis niloticus* (proposed model), Gene expression

## Abstract

**Background:**

Teleost fish skin is a vital barrier for regulating osmoregulation, immune defense, and respiration. Cutaneous wounds in farmed fish cause significant economic loss and welfare concerns, as standard treatments, like antibiotics and chemical baths, are often ineffective, toxic, and fail to adhere to mucus-covered surfaces. While nanomaterials show great promise for wound healing, current data on how they work in fish remain incomplete. This systematic review evaluates the performance of these nanomaterials, explains their healing mechanisms, and highlights the key research gaps to guide future aquaculture treatments.

**Results:**

Following PRISMA 2020 guidelines, we included twelve in vivo studies (2017–2026). A clear shift was observed over time: research before 2020 focused only on metallic nanoparticles (silver and zinc oxide), whereas recent studies have mostly used biopolymeric nano-delivery systems. Nanoparticle treatments consistently led to faster wound closure, with several formulas healing over 90% of the wound within two weeks; however, these quantitative outcomes are strictly descriptive due to high methodological heterogeneity across studies. Under the microscope, nanomaterials accelerated re-epithelialization, improved collagen organization, and reduced inflammation. At the molecular level, they decreased pro-inflammatory cytokines (*IL1β*,* TNFα*) and matrix metalloproteinases (*MMPS*), while increasing anti-inflammatory markers (*IL10*,* TGF-β)* and antioxidant enzymes (SOD, CAT). Transcriptomics data also showed the activation of fibroblast growth factor 7 (FGF7) and the Jak-STAT pathways. Finally, zebrafish (*Danio rerio*) constituted 75% of the study models, showing a major gap in research on farmed fish like Nile tilapia (*Oreochromis niloticus*).

**Conclusions:**

This systematic review shows that nanomaterials significantly accelerate teleost wound healing by promoting tissue regeneration and regulating the immune response. The shift toward biopolymeric systems, especially chitosan, indicates a clear trend toward safe and sustainable aquaculture medicine. Notably, testing hybrid nano-hyaluronic acid and nano-chitosan systems remains completely unexplored in farmed fish. This gap is important due to the complementary biological properties of these materials and the high global value of Nile tilapia. Testing this hybrid biomaterial under real farming conditions will help improve skin healing, support the immune system, and provide a safe wound treatment for global aquaculture.

**Supplementary Information:**

The online version contains supplementary material available at https://doi.org/10.1186/s12917-026-05934-6.

## Background

**Fig. 1:**
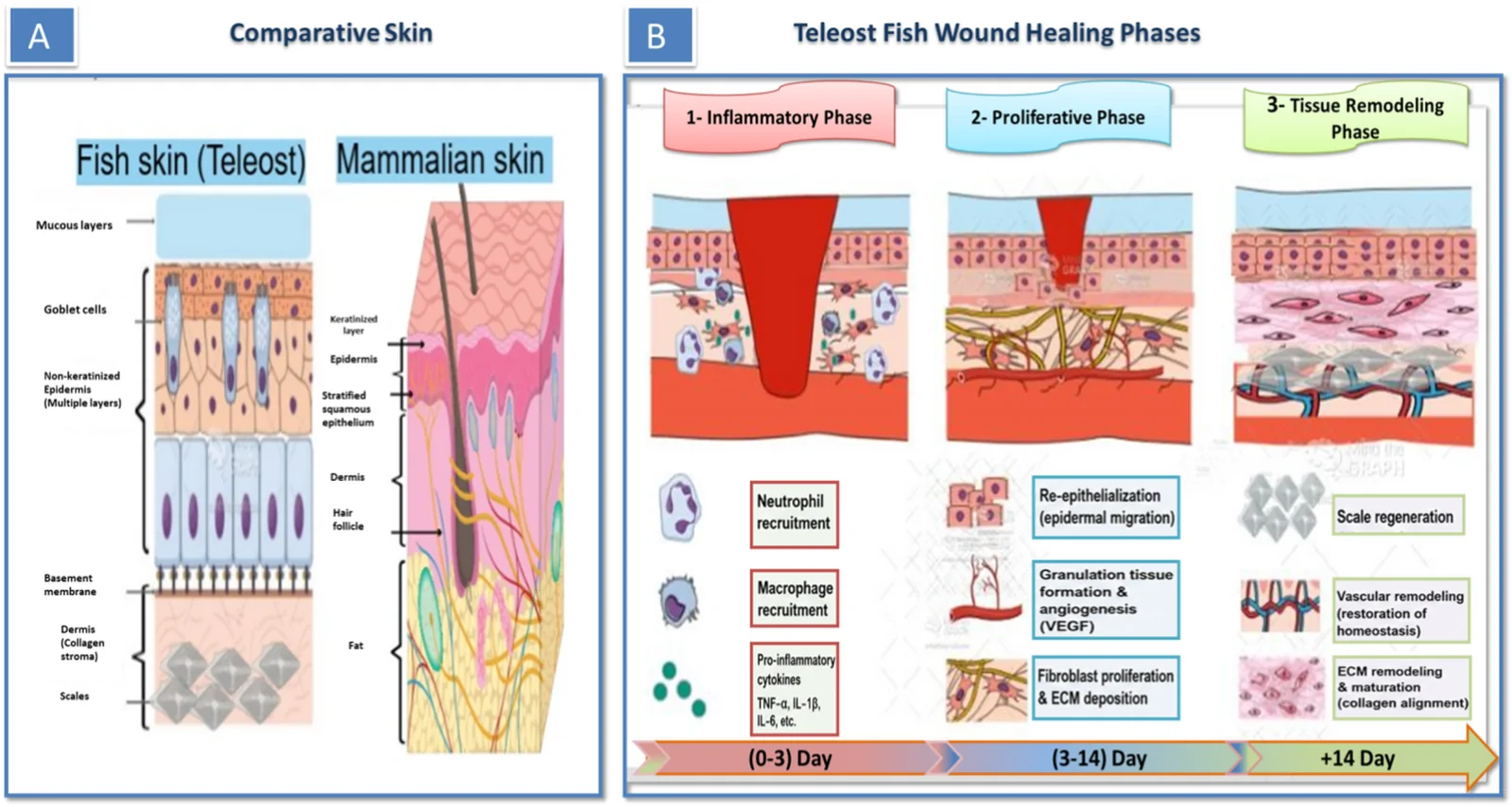
Schematic diagram of skin anatomy and wound healing phases. **A** A cross-section comparing teleost fish skin with mammalian skin. **B** The phases of wound healing in teleost fish. Image created with Mind the Graph (mindthegraph.com)

The skin is the largest organ of vertebrates and serves as the primary physical barrier protecting against dehydration, along with infectious and non-infectious environmental hazards [1]. For teleost fish, this organ is vital for aquatic survival, since it directly regulates osmoregulation, excretion, respiration, and immune defense [2]. Although fish skin is structurally similar to mammalian skin regarding basic components, it features unique structural variations that directly affect wound healing (Fig. 1A) [3]. Teleost skin consists of an epidermis and a dermis separated by a basement membrane. However, unlike mammalian skin, fish epidermis is non-keratinized, multi-layered, and continuously covered by protective, antimicrobial mucus secreted by goblet cells [4]. The dermis consists of a collagenous stroma and scales that originate from mesenchymal condensations within the dermal papillae [5, 6]. These unique anatomical features fundamentally shape how teleost fish respond to skin injuries and repair tissue [7, 8].

The process of cutaneous wound healing in teleost fish occurs in sequential and overlapping stages, with three distinct phases including the inflammatory, proliferative, and remodeling phases (Fig. 1B) [9]. Firstly, the rapid recruitment and accumulation of neutrophils and macrophages occur to clear debris and prevent infection in the inflammatory phase [10]. Following this, re-epithelialization, granulation tissue formation, angiogenesis, and deposition of collagen occur in the proliferative phase. Finally, the remodeling phase is characterized by the temporary continuation of extracellular matrix deposition at the wound edges to promote contraction and functional restoration [11].

In teleost models such as the Nile tilapia and zebrafish (the most widely studied species), re-epithelialization starts almost immediately after a wound occurs. Because the epidermal keratocytes are entirely alive and active, they can immediately migrate to close the wound. This fast response happens even before the inflammation reaches its peak, which is completely different from how mammalian skin heals [12, 13].

Fish wound repair can be assessed macroscopically and microscopically. Key methods include wound healing percentage (WHP), wound healing rate (WHR), and histopathological evaluation using H&E staining. Collagen assessment utilizes Masson’s trichrome staining, while molecular profiling examines cytokines and growth factors [14, 15].

Although aquaculture is growing rapidly, high-density farming often increases the incidence of physical injuries, parasitic infestation, and infectious diseases that damage the fish skin barrier and actively delay tissue repair [16]. These skin wounds in fish present a major challenge for both animal welfare and farm economics because they act as entry points for secondary pathogens and disrupt osmoregulation, thus leading to higher mortality rates and lowering the market value of the fish [17]. Traditional wound management, such as antibiotics and antiseptic bath treatments like hydrogen peroxide often yields ineffective or even toxic results, sometimes causing higher mortality in compromised fish [18]. Furthermore, these methods fail to protect the wound from the aquatic environment, lack bioactive properties to stimulate cellular regeneration, and can increase the risk of antimicrobial resistance [19]. Additionally, many of these treatments are impractical for large-scale aquaculture because they are difficult to apply. Handling fish individually induces severe stress and further mucus loss, and standard topical treatments do not adhere well to the wet, mucus-covered skin of fish [9].

The application of nanotechnology is considered as a highly effective approach for managing wounds in aquaculture [20]. Nano-scale materials (below 100 nm in at least one dimension) have unique physical and chemical properties due to their small size and large surface area [21]. Nanomaterials are highly effective for wound healing due to their ability to provide antimicrobial, anti-inflammatory, antioxidant, and pro-angiogenic effects, while also acting as delivery vehicles for bioactive molecules [22]. Nanomaterials offer practical applications in fish research and aquaculture due to diverse administration methods, including direct topical application, water immersion baths, or oral delivery via feed and gavage [23, 24].

Among the various nanoparticles, metallic and metal-oxide nanoparticles such as silver (AgNPs) and zinc oxide (ZnO NPs) are widely studied in wound healing due to their antimicrobial properties and ability to modulate reactive oxygen species (ROS) [25, 26]. Recently, research has focused on biopolymer-based nanomaterials, including chitosan nanoparticles, hyaluronic acid derivatives, liposomal formulations, and nanoencapsulated natural extracts to improve wound healing [27, 28]. These biopolymers are useful because they are biocompatible, biodegradable, and provide a structure that resembles the extracellular matrix (ECM) to support tissue regeneration [29]. Within these biopolymers, the natural polysaccharide chitosan acts as a hemostatic and antimicrobial agent that easily adheres to wet mucosal surfaces. Additionally, hyaluronic acid is a major ECM component that maintains tissue hydration and creates a framework for cells to migrate and heal the wound [30, 31].

Based on the current literature, there is a major gap in applying advanced biomaterials to aquaculture. While nanoparticles like silver, zinc oxide, and nano-chitosan have been studied for fish wound healing [32], nano-hyaluronic acid remains largely unexplored in teleost models. Furthermore, to the best of our knowledge, no studies have evaluated the combined therapeutic effects of nano-hyaluronic acid and nano-chitosan. Currently, most studies depend on invasive sampling and basic histology, without using non-invasive tools like ultrasound. Moreover, advanced tissue evaluations using Masson’s trichrome for collagen, immunohistochemistry (IHC), and scanning electron microscopy (SEM) are rarely performed. This research gap is evident even in major teleost models, such as the zebrafish (a biological model for in vivo wound healing research) [13] and the Nile tilapia (*Oreochromis niloticus)*, an economically vital farmed species yielding over 6.5 million tons globally annually [33].

The rationale for exploring the combination of hyaluronic acid and chitosan provides biological and physical advantages for fish wound healing. Biologically, chitosan provides antimicrobial and hemostatic effects, while hyaluronic acid regulates inflammation, stimulates angiogenesis, and supports cell migration [34, 35]. Physically, native hyaluronic acid is highly hydrophilic and cannot form stable nanoparticles alone [36]. Although chemical crosslinking can be toxic, mixing hyaluronic acid with positively charged chitosan creates stable, crosslinker-free nanogels through electrostatic attraction. This natural interaction prevents drug leakage and ensures safe delivery to fish skin [37].

This systematic review aims to integrate basic biomaterial science with practical fish farming. Our objectives are to evaluate how nanoparticle biomaterials heal skin wounds in fish, analyze the underlying cellular, microstructural, and molecular mechanisms, examine publication trends over time, and assess the quality of studies. By identifying these gaps, this work provides the scientific basis to develop combined hyaluronic acid-chitosan nanomaterials treatments for fish wound healing, especially for economically important species like the Nile tilapia.

## Methodology

### Protocol registration

The protocol for this systematic review was retrospectively registered on the Open Science Framework (OSF) platform (https://osf.io/kz5ga*)* on July 16, 2026.

### Study design and PRISMA compliance

From April to May 2026, a systematic review was conducted following the Preferred Reporting Items for Systematic Reviews and Meta-Analysis (PRISMA 2020) guidelines, with the SYRCLE (Systematic Review Centre for Laboratory Animal Experimentation) principles considered for in vivo animal model methodology. The primary objective was to evaluate the efficacy of different nanoparticle-based biomaterials in promoting cutaneous wound healing in fish models.

### Research questions and scope

The review addressed three core questions: (1) Therapeutic efficacy: what is the effect of nanoparticle-based biomaterials on the cutaneous wound closure rate and the histopathological healing stages (inflammation, proliferation, and tissue remodeling) in fish models? (2) Pathological and molecular mechanism: What are the effects of these nanomaterials on tissue architecture, as assessed by staining (e.g., H&E, Masson’s trichrome), ultrastructural imaging (e.g., SEM), and how do they alter the expression of immune and growth-related genes (e.g., TNF-α, IL-1β, TGF-β)? (3) Comparative performance: Does nanoparticle treatment significantly improve tissue regeneration and morphological recovery compared to untreated controls or conventional therapies?

### Search strategy and data sources

A literature search was conducted using PubMed, Scopus, and Web of Science. The search strategy used a combination of Medical Subject Headings (MeSH) and keywords connected by Boolean operators (AND, OR), as follows: (“aquaculture” OR “fish” OR “teleost” OR “Nile tilapia” OR “*Oreochromis niloticus*”) AND (“chitosan” OR “nano-chitosan” OR “hyaluronic acid” OR “hyaluronan” OR “albumin” OR “nanoparticle” OR “nanomaterial”) AND (“wound healing” OR “tissue regeneration” OR “skin repair” OR “wound repair”) AND (“histopathology” OR “pathology” OR “immunohistochemistry” OR “IHC” OR “SEM” OR “masson”)). Reference lists of retrieved articles were also manually screened for relevant studies.

### Eligibility criteria

The inclusion and exclusion criteria were established based on the PICO framework. To ensure relevance, only articles published between 2017 and 2026 were considered.

#### Inclusion criteria

1. Population (P): In vivo fish models (teleost) with experimentally induced cutaneous wounds.
2. Intervention (I): Application of nanoparticle-based biomaterials for wound healing (e.g., as dressings, ointments, or via water immersion).
3. Comparison (C): Untreated control groups (negative control) or conventional (positive control) therapies.
4. Outcome (O): Macroscopic wound closure rates, histopathological healing parameters (e.g., re-epithelialization), and immune-related gene expression.
5. Study Type: Original, peer-reviewed in vivo research articles published in English.

#### Exclusion criteria

- Studies conducted on mammalian models (e.g., mice, rats, human) or non-fish species.
- Intervention lacking nanomaterial or utilizing fish-derived materials applied to non-aquatic animals.
- Studies focusing on non-cutaneous tissue engineering (e.g., bone) or strictly on in vitro models.
- Secondary literature (review articles, meta-analyses), conference abstracts, and book chapters.

### Study selection process

All search results were exported to Rayyan [38] for semi-automated duplicate removal. Two independent reviewers (A.R. and I.I.) screened the titles and abstracts based on the PICOS criteria. Subsequently, the full texts of eligible articles were assessed. To minimize publication bias, the reference lists of included studies were manually cross-referenced. Any disagreements were resolved by a third reviewer (H.M. or A.E.).The final numerical selection process is detailed in the Results section and summarized in the PRISMA 2020 flowchart.

### Data extraction and synthesis

Data extraction into a standardized Microsoft Excel spreadsheet was performed independently by two reviewers (A.R. and I.I.), and all extracted data were cross-checked. Any discrepancies were resolved through discussion or consultation with a third reviewer (H.M. or A.E.). For each included study, the following variables were collected:

- Study Characteristics: First author and year of publication.
- Fish model details: Fish species, animal size/weight.
- Wound characteristics: Type, size, and anatomical location of the experimentally induced wound.
- Intervention: Type of nanoparticle, synthesis method, dosage/concentration, and application route.
- Comparison: control groups utilized (untreated or traditional therapies).
- Macroscopic outcomes: wound retraction and overall closure rates.
- Microscopic, immunohistochemical, and molecular outcomes: Histopathological evaluations using H&E staining (e.g., for re-epithelialization and inflammation), Masson trichrome staining for collagen deposition, SEM, IHC, and immune-related gene expression (e.g., *TNF-α.*,* IL-1β*,* TGF-β*).

Due to the variability in nanoparticle types, Fish species, and experimental protocols, a meta-analysis was not possible. Instead, we performed a narrative synthesis according to the Synthesis Without Meta-analysis (SWiM) guidelines [39]. Data were tabulated and qualitatively analyzed to evaluate pathological outcomes.

### Quality assessment (Risk of bias)

We assessed the risk of bias in the included studies using SYRCLE’s tool [40], which is adapted from the Cochrane tool for animal studies. Two independent reviewers checked each study across ten domains covering six types of bias: selection, performance, detection, attrition, reporting, and other biases. Any disagreements were resolved by discussion or a third reviewer. We rated the risk in each domain as low, high, or unclear, and created the final plots using the *robvis* tool [41].

### **AI use**

Gemini was used to improve grammar and clarity. The authors reviewed the text and take full responsibility for the content.

## Results

### Study selection

**Fig. 2:**
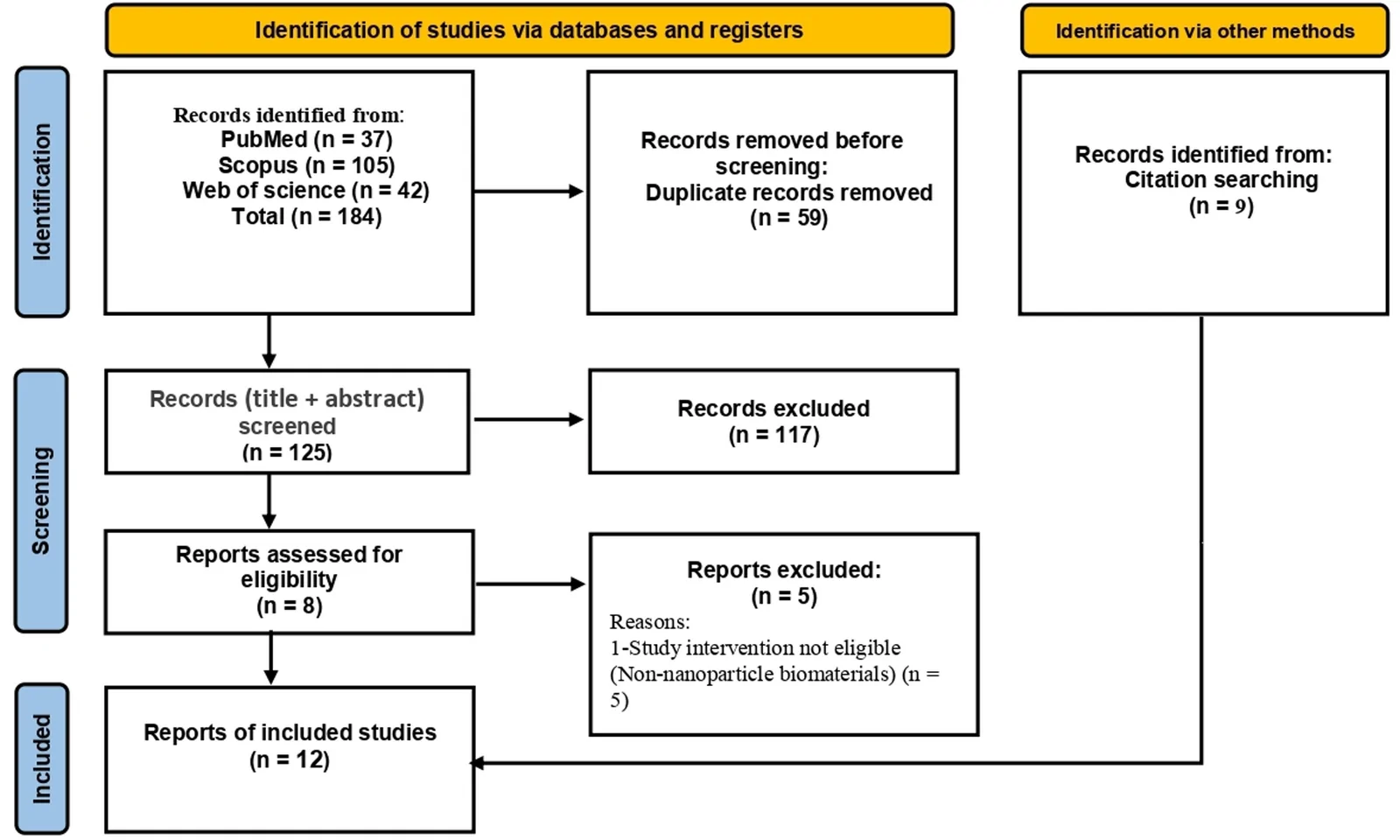
PRISMA flowchart of study selection

Our systematic search across PubMed, Scopus, and Web of Science from April to May 2026 initially found 184 records (37 from PubMed, 105 from Scopus, and 42 from Web of Science). After removing 59 duplicates, we screened the titles and abstracts of the remaining 125 studies and excluded 117 studies that did not meet our criteria. We then reviewed the full text of the remaining 8 studies and excluded 5 for using non-nanoparticle materials (such as standard collagen dressings or regular chitosan), leaving 3 eligible articles. Additionally, 9 relevant studies were identified by manually searching reference lists. In total, 12 original in vivo studies met all criteria and were included in this review. The entire selection process is illustrated in the PRISMA 2020 flow diagram (Fig. 2).

### Research trends

**Fig. 3:**
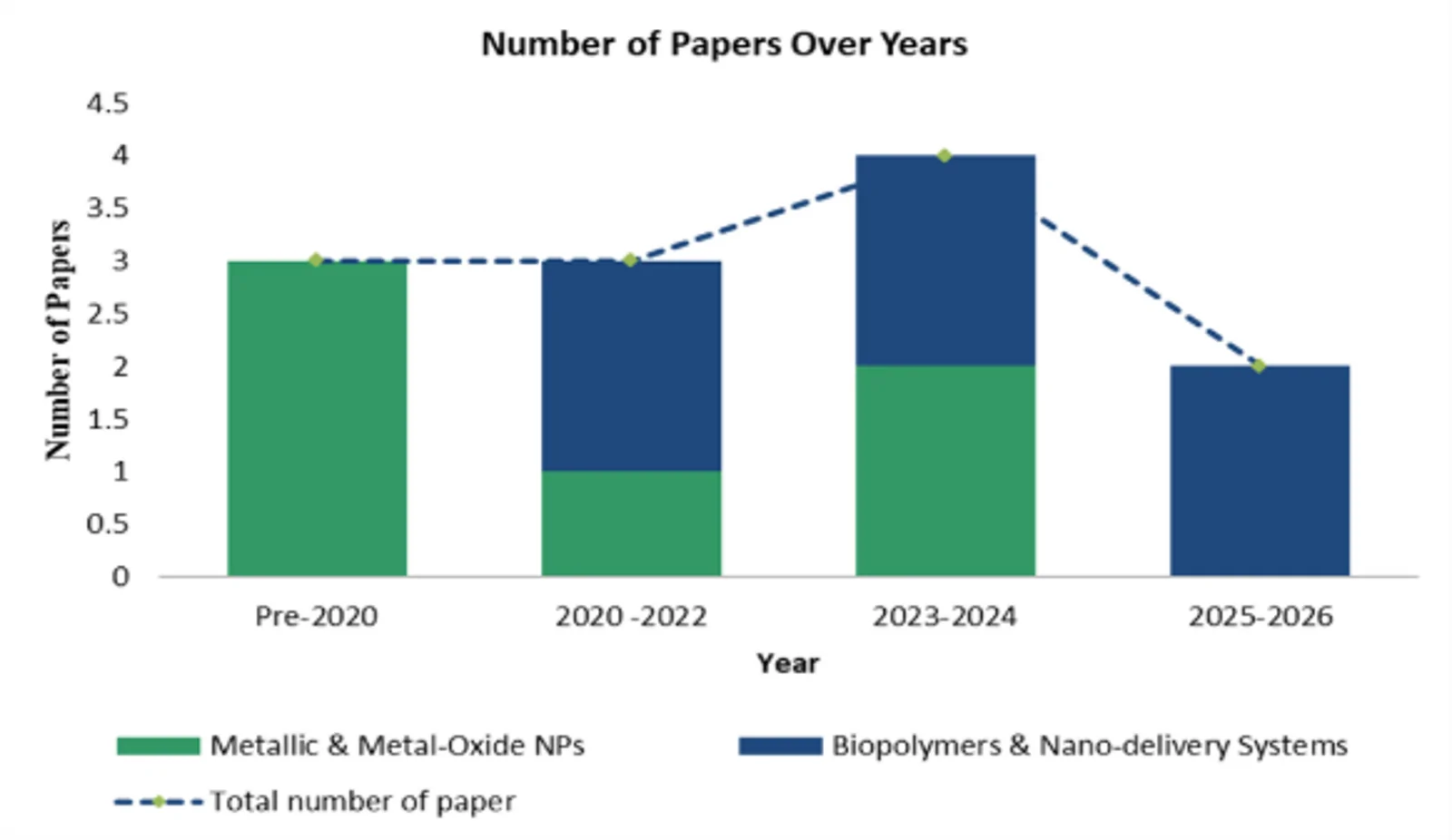
Research trends and the shift from metallic to biopolymeric nanomaterials in fish wound healing

Our timeline analysis of the 12 included studies reveals a clear shift in fish wound healing research from metallic nanoparticles to natural biopolymers (Fig. 3). Before 2020, the three published studies focused mainly on metallic nanoparticles, specifically silver or zinc oxide (AgNPs or ZnO NPs), with one study combining silver with chitosan. Between 2020 and 2022, the trend started to change, with two out of three studies using biopolymeric and nano-delivery systems like chitosan, pectin nanoparticles, and nanoemulsions. Research surged between 2023 and 2024 with four studies, equally split between metallic and biopolymeric systems. Notably, the two studies published in the recent 2025–2026 period focused mainly on biopolymers and nano-delivery systems, specifically octominin-chitosan nanoparticles and resveratrol-loaded liposomes, with no metallic nanoparticle research. This trend highlights that the scientific field is moving toward biocompatible materials, supporting our goal to develop a hyaluronic acid-chitosan system for fish wound care.

### Features of the included studies

The 12 included in vivo studies used different fish models to evaluate wound healing (Table 1). Zebrafish (*Danio rerio*) was the most common species, appearing in 9 of the 12 studies (75%) [14, 15, 42–48], mainly because of their well-characterized genome and optical transparency. The other three studies used Mozambique tilapia *(Oreochromis mossambicus)* [49], goldfish *(Carassius auratus)* [50], and Asian catfish *(Pangasius hypophthalmus)* [51]. Researchers induced wounds in several ways. Mechanical wounds made with biopsy punches or scalpels were the most frequent (*n* = 7) [15, 43, 45, 46, 48, 49, 51], followed by laser ablation (*n* = 3) [14, 44, 47], fin amputation (*n* = 2) [42, 45], and natural parasitic lesions (*n* = 1) [50]. To apply the nanomaterials, most studies simply added them to the water as immersion baths (*n* = 7) [14, 42, 45, 46, 48–51]. Others applied the treatments topically directly to the wound (*n* = 4) [14, 15, 43, 44], and one study gave the treatment orally (*n* = 1) [47]. The experiments monitored the healing process for anywhere from 10 to 50 days. To evaluate healing, 11 studies used macroscopic analysis, mostly through digital photography and ImageJ software to calculate the wound healing percentage (WHP) or closure rate [14, 15, 42–50]. Among these, one study relied only on tabulated measurements without photographs [51], while another used in vivo fluorescence microscopy to track tissue regeneration and cell recruitment [42]. For microscopic evaluation, eight studies used H&E staining to monitor tissue repair [14, 15, 43, 44, 46, 49–51].To study healing at the molecular level, seven studies analyzed gene expression using qRT-PCR, with four focusing on wound healing-related genes (including inflammatory and regenerative markers) [15, 43, 45, 46] and three evaluating both wound healing and antioxidant genes [14, 44, 48]. Additionally, one used RNA-seq transcriptomics combined with a DHE assay to measure in vivo ROS scavenging [42].

**Table 1:** General characteristics of the included in vivo studies on nanomaterials for fish wound healing

Study	Fish Model	Wound Details	Intervention	Duration	Control Groups	Evaluation Methods
Seo et al. [14]	Zebrafish (*Danio rerio*), wild-type (4 months old)	Laser-induced, single wound, laser beam (150 mA for 5 s) (posterior to gill area)	AgNPs (Chemical reduction); (direct application) 2 mg/wound directly on the site outside water for 4 min (then washed for 5 min in a separate tank) or (immersion) 50 mg/L in a separate solution for 4 min (Both applied on 0, 2, 5, and 7 dpw	20 days (Sampling: 2, 5, 7, 10, 14, 20 for histology dpw; Gene exp.: 12hpw, 24hpw, 5 dpw)	1. Wounded-untreated (Positive Control) 2. Non-wounded (Negative Control)	Macroscopic: WHP via ImageJ. Microscopic: H&E. Molecular: qRT-PCR (*TGF-β*,* IL-1β*,* TNF-α*,* MMP-9*,* MMP-13*, SOD, CAT).
Abu-Elala et al. [50]	Goldfish (*Carassius auratus*), fingerlings (12.8 ± 2.2 g)	Natural parasitic wounds ( ulcerative skin and muscle lesions from *Lernaea cyprinacea*)	Chitosan-silver nanocomposites; dose: 5.495 ppm (aqueous immersion for 24 h) then transferred to fresh water	14 days (24 h exposure + 2 weeks monitoring)	1. Wounded-untreated (Positive Control): infested 2. Unwounded (Negative Control): Healthy, non-infested	Macroscopic: Clinical monitoring. Microscopic: H&E.
Ravindran et al. [51]	*Pangasius hypophthalmus* (15–25 g, 8–13 cm)	Excisional wound via biopsy punch needle (belly region)	Green AgNPs (synthesized via 1 mm AgNO_3_ and T. procumbens extract) via aqueous exposure	10 days (Wound area every 2 days; Histology sampling every 5 days (days 0, 5, 10)	1.Wounded untreated, 2.AgNO₃ alone, 3.Plant extract alone	Macroscopic: Wound area measurement & healing percentage (Tabulated data; no macro photos). Microscopic: H&E.
Rajapaksha et al. [44]	adult wild-type (AB strain) Zebrafish (*Danio rerio*), (4 months old, (0.45 ± 0.05 g)	Laser-induced full-thickness dermal wound (150 mA for 5 s) created on a dark stripe at (the left flank anterior to the anal and dorsal fins).	SmPNPs (Spirulina maxima-derived pectin nanoparticles); (topical application) on the wound site at a dose of 600 µg/fish (dosed at 0, 1, 2, 4, 6, and 10 dpw)	24 days (Wound closure & pigment imaging: 2, 7, 10, 14, and 24 dpw; Histology (H&E): 2 and 7 dpw; Gene expression (qPCR): 1, 2, 7, 14, and 24 dpw.	1. Wounded-untreated (Positive Control): Vehicle (Wounded +(ddH_2_O)) 2. Unwounded (Negative Control)	Macroscopic: (WHP), (WHR), and pigment restoration. Microscopic: H&E. Molecular: qRT-PCR (*TGF-β*,* IL-1β*,* TNF-α*,* MMP-9*,* MMP-13*, and).
Zain et al. [48]	Zebrafish (*Danio rerio*), adult wild-type AB strain (0.40 ± 0.05 g)	biopsy punch-induced circular wound, 2 mm diameter (posterior to gill area)	1-OPL-FEE: 6.25 mg/L 2-OPL-FEE-NE 0.5 g/L (immersion) (applied every 2 days)	30 days (Sampling: wound closure: 2, 4, 8, 11, 20, and 30 dpw; Gene expression (qRT-PCR): 2 and 8 dpw)	1. Wounded Untreated (Negative control). 2. Allantoin-treated Wounded (Positive control). 3. Non-wounded (Baseline reference)	Macroscopic: Wound closure via ImageJ. Molecular: qRT-PCR (*TGF-β*,* IL-1β*,* TNF-α*,* MMP-9*,* MMP-13*, *TIMP*2b, SOD1, CAT).
Iswarya et al. [49]	Mozambique tilapia (*Oreochromis mossambicus*), fingerlings (10.25 ± 0.3 cm, 9.36 ± 0.3 g)	metallic circular biopsy puncher, 3 mm diameter (above lateral line)	Phβ-GBP-AgNPs (Biological synthesis); 400 µg/L (immersion) chem-AgNPs (Chemical synthesis); 300 µg/L (immersion)	20 days (Sampling: every 4 days; Histology: 3-day intervals for 2 weeks)	1. Control-non-infected (untreated) 2. Control-infected (untreated + A. hydrophila) 3. Infected + Phβ-GBP alone (100 µg/L) 4. Infected + chem-AgNPs (300 µg/L) 5. Infected + Phβ-GBP-AgNPs (400 µg/L)	Macroscopic: WHP via ImageJ. Microscopic: H&E.
Cao et al. [42]	Zebrafish (*Danio rerio*) Adult (5 months old): Wild-type and Transgenic line (coro1a: EGFP to track leukocytes). Larvae/Embryo (24 hpf): Wild-type and Double Transgenic line (fli1: EGFP/gata1: mCherry to track blood vessels/RBCs).	1. Physical Wound (Adults): Caudal fin amputation using a scalpel 2. Chemical Vascular Injury (Larvae): Exposure to 0.1% H_2_O_2_.	Nano-Se@S; Dose: 8 ng/mL (consisting of 4 ng/mL Nano-Se + 4 ng/mL Nano-S). ( immersion)	Fin Regeneration: 8 days. RNA Sampling: 10 days. Inflammation Tracking: 48 h. Vascular Injury/ROS: 20 min.	1. Amputated-untreated (Control in system water/PBS). 2. Nano-Se alone (4 ng/mL). 3. Nano-S alone (4 ng/mL)	Macroscopic: Fin regeneration rate (Regeneration length / Resection length × 100%) [Adult Model] Macroscopic: Fluorescence microscopy 1-Tracking leukocyte recruitment at the wound site [Adult Model]. 2-Observing angiogenesis (sprouting subintestinal veins) and evaluating vascular protection against H2O2-induced damage [Larvae Model Molecular: RNA-Seq (Transcriptomic analysis) of regenerated fin tissue (identifying pathways like FGF via FGF7 and Jak-STAT) [Adult Model]. Biochemical: Use of DHE (dihydroethidium) fluorescent probes to detect and quantify in vivo ROS (superoxide) scavenging [Larvae Model].
Ramachandran et al. [45]	Adult zebrafish (*Danio rerio*), AB strain (6–8 months)	Standardized amputation (dorsal fin) via scalpel	Ch-ZnO-NPs; 30 µg/mL via continuous immersion	15 days (wound closure Imaging at 3, 7, and 15 dpw; Gene expression (qPCR) at 15 dpw).	1. Wounded-untreated (Positive Control): ( 0.05% v/v PBS) 0.2. β-chitosan alone	Macroscopic: Wound closure (%) via ImageJ (based on stereomicroscopy images). Molecular: RT-PCR (*Bax*,* BCl-2*,* IL-2*,* IL-6*,* TNF-α*).
Tayyeb et al. [46]	Zebrafish (*Danio rerio*), adult wild-type	Scalpel-induced incision (behind gill region, near lateral line)	ZnO-CN NPs (Chemical synthesis); 40 and 80 µg/mL via immersion (applied every third day	14 days (Sampling: 0, 7, and 14 dpw for Wound sampling, Histology & Gene expression:	1. Wounded-untreated (Positive Control) 2. Non-wounded (Negative Control)	Macroscopic: Wound closure via ImageJ. Microscopic: H&E. Molecular: qRT-PCR (*IL-1β*,* TNF-α*,* MMP-13*).
Kandaswamy et al. [43]	Adult zebrafish (*Danio rerio*), wild type (10–12 months)	Full-thickness puncture wound (posterior to the gill region near the lateral line) via scalpel	Cu@Bbc loaded PCL Nanofibers (Combinatorial Curcumin: (240 µg/ml) and Berberine chloride: (200 µg/ml) encapsulated in 15% Poly (€-caprolactone) nanofibers via electrospinning ; applied topically on the wound site every third.	12 days (Wound closure imaging at 0, 3, 6, 9, and 12 dpw; Histopathology and qPCR: Taken during/at the end of the study).	1. Wounded-untreated (Positive Control): 2. Curcumin alone (240 µg/ml) 3. Berberine chloride alone (200 µg/ml)	Macroscopic: ImageJ closure. Microscopic: H&E Molecular: qPCR (*IL-1β*,* TNF-α*,* MMP-13*).
Dias et al. [15]	Zebrafish (*Danio rerio*), adult (4 months old, 0.45 ± 0.05 g)	Biopsy punch-induced circular wound, 3 mm diameter (between anal and dorsal fins)	Octominin-CNPs (Ionotropic gelation); 20 µg/wound (topical application : outside water on moist gauze for 4 min) (applied on days: 0, 2, and 4 dpw)	21 days (Sampling: Wound closure sampling: 2, 4, 7, 14, and 21 dpw ; histology: 7 dpw only; Gene expression (qRT-PCR): 2 and 7 dp	CNPs alone, Octominin alone 1. Wounded-untreated (Positive Control): Wounded + 1× PBS. 2. Non-wounded (Negative Control) 3. CNPs alone-treated Wounded 4. Octominin alone-treated Wounded	Macroscopic: WHP and WHR via ImageJ .Microscopic: H&E. Molecular: qRT-PCR (*IL-1β*,* TNF-α*,* IL10*,* cxcl18b*,* ccl34a4*,* MMP9*,* MMP13*).
Yu et al. [47]	Adult zebrafish (*Danio rerio*), 4–6 months old	Full-thickness Co_2_ laser-induced ablation wound (~ 400 μm deep, 2 mm diameter) (on the right flank anterior to the anal and dorsal fins).	Resveratrol-loaded liposomes (PC-RSV); ~200 nm; 1 mg/kg via oral gavage (dosed twice weekly).	50 days (Wound closure imaging (WC%): Every 5 days at 5, 10, 15, 20, 25, 30, 35, 40, 45, 50 dpi)	1.Wounded-untreated (Positive Control): Orally dosed with sterile (ddH_2_O), 2.Blank PC liposome 3. Blank PS liposome.	Macroscopic: Wound closure (%) via ImageJ (using dissecting microscopy images

*AgNO₃* Silver nitrate, *AgNPs* Silver nanoparticles, *Bax* Bcl-2 associated X protein, *Bcl-2* B-cell lymphoma 2, *CAT* Catalase, *ccl34a4* Chemokine (C-C motif) ligand 34a4, *chem-AgNPs* Chemically synthesized silver nanoparticles, *CNPs* Chitosan nanoparticles, *Cu@Bbc* Curcumin-berberine chloride, *cxcl18b* Chemokine (C-X-C motif) ligand 18b, *ddH₂O* Double-distilled water, *dpi* Days post-injury, *dpw* Days post-wounding, *EGFP* Enhanced green fluorescent protein, *FGF* Fibroblast growth factor, *H&E* Hematoxylin and eosin staining, *H₂O₂* Hydrogen peroxide, *hpf* Hours post-fertilization, *IL* Interleukin, *ImageJ* Image processing and analysis software, *Jak-STAT* Janus kinase/signal transducers and activators of transcription, *MMP* Matrix metalloproteinase, *Nano-Se@S* Selenium-sulfur core-shell nanoparticles, *NC* Negative control, *OPL-FEE* 1-Octyl-D-Phenylalanine-functionalized *Euphorbia hirta* ethanolic extract, *OPL-FEE-NE* 1-Octyl-D-Phenylalanine-functionalized *Euphorbia hirta* ethanolic extract nanoemulsion, *PBS* Phosphate-buffered saline, *PC* Phosphatidylcholine, *PCL* Poly(ε-caprolactone), *PC-RSV* Phosphatidylcholine-resveratrol loaded liposomes, *Phβ-GBP* *Phyllanthus emblica* beta-glucan binding protein, *ppm* Parts per million, *PS* Phosphatidylserine, *qRT-PCR / RT-PCR* Quantitative reverse transcription polymerase chain reaction, *RNA-Seq* RNA sequencing, *SmPNPs* *Spirulina maxima*-derived pectin nanoparticles, *SOD* Superoxide dismutase, *TGF-β* Transforming growth factor beta 1, *TIMP2b* Tissue inhibitor of metalloproteinase 2b, *TNF-α* Tumor necrosis factor alpha, *WHP* Wound healing percentage, *WHR* Wound healing rate, *ZnO-CN NPs* Zinc oxide-chitosan nanoparticles

### Macroscopic and microscopic outcomes

#### Macroscopic wound healing

All 12 studies reported macroscopic wound healing outcomes. Quantitative data demonstrated that nanoparticle-treated groups recorded higher wound closure rates compared to untreated or vehicle controls (Table 2). However, due to the high methodological heterogeneity in nanomaterial types, doses, wound models, and species across the included studies, these quantitative outcomes are presented descriptively and should not be directly compared.

**Table 2:** Macroscopic and microscopic wound healing outcomes

Author	Analysis	Overall Result
Dias et al. [15]	Macroscopic analysis	Octominin-CNPs (at 21 dpw): higher WHP (91.73 ± 1.56%)^*^ and peak WHR (1.50 ± 0.10)^*^ compared to vehicle. Vehicle Group (at 21 dpw): Showed lower WHP (77.35 ± 2.88%). 4 dpw: Initial wound area expansion observed due to inflammatory response. 21 dpw: Complete wound closure, resolved margins, and increased pigmentation across groups.
	microscopic evaluation	Re-epithelialization & Muscle Repair: Octominin-CNPs showed faster healing and better muscle tissue repair at 7 dpw. Inflammation: Much lower inflammation compared to vehicle and raw Octominin groups. Tissue necrosis: marked reduction in tissue necrosis and excessive granulation layers at 7 dpw.
Seo et al. [14]	Macroscopic analysis	Wound Closure (Day 20): All AgNPs-treated fish showed complete wound healing with no visible marks. Immersion Method: Showed the best results, with a WHP of 36.6%^*^ on day 5 and 78.8%^*^ on day 14. Topical Method: Showed a WHP of 23.7%^*^ (day 5) and 74.8%^*^ (day 14), which was higher than the untreated control (18.2% and 68.3%). Wound Shape (Laser): Started with dark skin and mild hemorrhage at 0 h, and then formed clear measurable edges by day 2.
	Microscopic evaluation	Tissue Repair (Day 5): complete filling of the wound cavity compared to deep untreated wounds. Skin Structure: Induced a thick epithelium layer, dense dermal layer, and well-organized granulation tissue. Immersion Group: Enhanced early differentiation of epidermal cells into well-formed skin, immune cell aggregation at wound margins, and skeletal muscle regeneration (while control groups showed damaged muscle fibers).
Iswarya et al. [49]	Macroscopic analysis	Phβ-GBP-AgNPs (at 16 dpw): higher WHR (92%)^*^ compared to (61.2%) Phβ-GBP, (74.8%) chem-AgNPs, (48.3%( non-infected control, and (33.4%) infected control). 4 dpw; Blood vessel formation observed. Pigmentation began at the wound edges on day 8dpw and complete closure by 16 dpw.
	Microscopic evaluation	The wound cavities complete closure with newly formed skin and scales at 16 dpw.
Tayyeb et al. [46]	Macroscopic analysis	At 7 dpw: Wound closure reached 54%^*^ at 40 µg/mL and 62%* at 80 µg/mL. At 14 dpw: Wound closure reached 70%^*^ at 40 µg/mL and 93%^*^ at 80 µg/mL versus untreated control.
	Microscopic evaluation	At 14 dpw: complete tissue repair and full re-epithelialization. Tissue Structure: Regeneration of skeletal muscles, dermal layer, and dense granulation tissue. Epidermis & Cells: Complete epidermal layer formation with controlled inflammatory infiltration.
Zain et al. [48]	Macroscopic analysis	At 8 dpw: All treated groups surpassed 40% wound closure. At 11 dpw: OPL-FEE-NE reached 87%^*^ closure, which was higher than OPL-FEE extract (77%). At 20 dpw: All treated groups surpassed 80% wound closure. By 30 dpw: Complete wound healing achieved (skin color completely pigmented) with minimal scar formation.
	Microscopic evaluation	Not Evaluated.
Ramachandran et al. [45]	Macroscopic analysis	At 1 dpw: -βCh-ZnO-NPs group started showing early reduction in wound area compared to other groups. At 8 dpw: Peak healing efficacy was achieved; β-Ch-ZnO-NPs maintained 70%^*^ efficacy and β-Ch alone reached 60%^*^, significantly outperforming the untreated control (35%). By 15 dpw: Treatment efficacy diminished over time; β-Ch-ZnO-NPs decreased to 30%^*^ efficacy and β-Ch alone dropped to 15%^*^, while the untreated control reached 55%.
	Microscopic evaluation	Not Evaluated.
Kandaswamy et al. [43]	Macroscopic analysis	By 14 dpw: The Cu@Bbc nanofiber achieved 94%^*^ wound closure with no visible wounds, significantly higher than Bbc nanofiber at 83%, Cu nanofiber at 76%, and the untreated control group at 62%.
	Microscopic evaluation	at 14 dpw; Cu@Bbc Nanofiber: Showed a robust epithelial layer, advanced granulation tissue, restored muscle cells, and the lowest inflammation. Cu & Bbc Nanofibers: Showed only a thin epithelial layer, early-stage granulation, and partial muscle cell regeneration. Control Group: Completely lacked an epidermal layer and retained the highest accumulation of inflammatory cell.
Yu et al. [47]	Macroscopic analysis	25% Closure: Achieved at 10.2 dpi^*^ with PC-RSV liposome (vs. 16.9 dpi in Control). Significant wound size reduction observed by 10 dpi. 50% Closure: Achieved at 15.3 dpi^*^ with PC-RSV liposome (vs. 23.1 dpi in Control). 75% Closure: Achieved at 23.7 dpi^*^ with PC-RSV liposome (vs. 37.4 dpi in Control), with near-complete re-epithelialization by 20 dpi. Empty Liposomes (PC & PS): Showed no significant improvement over the control group,
	Microscopic evaluation	Not Evaluated.
Rajapaksha et al. [44]	Macroscopic analysis	By 24 dpw: Topical application of SmPNPs achieved rapid and complete wound closure, significantly outperforming the vehicle group at 10 dpw with 48.90%^*^ WHP (vs. 38.80%), and almost completely restored melanin-based skin pigments to an intensity area of 135.00 mm²^*^ (vs. 91.0 mm² in the vehicle group)
	Microscopic evaluation	By 7 dpw: SmPNPs-treated wounds exhibited complete re-epithelialization with a well-developed neoepidermis measuring 53 μm^*^ (vs. 18 μm in the vehicle group) and reduced inflammation, following accelerated granulation tissue formation at 2 dpw despite an initial increase in leukocyte infiltration.
Abu-Elala et al. [50]	Macroscopic analysis	By 1 week: Parasitic skin ulcers were fully healed and covered with a white membrane within one week after treatment.
	Microscopic evaluation	After treatment: Wounded tissue showed complete surface re-epithelialization, well-organized collagen fibers, and new blood capillaries with reduced inflammation.
Ravindran et al. [51]	Macroscopic analysis	By day 10: Wounds treated with AgNPs (T3) achieved the highest closure rate of 92.09%^*^ (reducing wound size from 1.77 cm to 0.14 cm), significantly outperforming the control (65.54%), AgNO3 (70.05%), and plant extract (77.96%) groups.
	Microscopic evaluation	Day 5: Wounds treated with AgNPs (T3) showed early collagen deposition and hemorrhage. Control exhibited persistent necrosis, inflammation, and granuloma formation. Day 10: T3 showed complete recovery of the epithelial architecture with well-organized mature collagen, whereas the control group exhibited persistent necrosis and no epithelialization, and other treatments (T1/T2) showed only partial healing.
Cao et al. [42]	Macroscopic analysis	By day 8: Based on the regeneration curve (Fig. 2E), 8 ng/mL Nano-Se@S significantly accelerated fin recovery, starting from 4 dpa (~ 10%^*^ vs. ~ 5% in control), reaching 6 dpa (~ 21%^*^ vs. ~ 13% in control), and peaking at 8 dpa (~ 26%^*^ vs. ~ 20% in control).
	Microscopic evaluation	Adults: Nano-Se@S significantly increased early neutrophil/macrophage recruitment at fin wound site 6 h^*^ post-injury (*coro1a: EGFP*). Larvae: Nano-Se@S stimulated angiogenesis by increasing subintestinal vein (SIV) sprouting after 48 h^*^ of treatment (*fli1: EGFP*). Larvae: Nano-Se@S protected against vascular injury (prevented torso vessel narrowing/malformation) induced by 0.1% H _2_ O _2_ for 20 min (*fli1: EGFP/gata1: mCherry*). Antioxidant Efficacy Larvae: Nano-Se@S significantly decreased superoxide levels in a dose-dependent manner (via in vivo DHE probe).

*AgNO₃* Silver nitrate, *AgNPs* Silver nanoparticles, *β-Ch-ZnO-NPs* Beta-chitin zinc oxide nanoparticles, *Cu@Bbc* Curcumin-berberine chloride, *dpa* Days post-amputation, *dpi* Days post-injury, *dpw* Days post-wounding, *DHE* Dihydroethidium, *EGFP* Enhanced green fluorescent protein, *H₂O₂* Hydrogen peroxide, *Nano-Se@S* Selenium-sulfur core-shell nanoparticles, *OPL-FEE* 1-Octyl-D-Phenylalanine-functionalized Euphorbia hirta ethanolic extract, *OPL-FEE-NE* 1-Octyl-D-Phenylalanine-functionalized Euphorbia hirta ethanolic extract nanoemulsion, *PC-RSV* Phosphatidylcholine-resveratrol loaded liposomes, *Phβ-GBP-AgNPs* Phyllanthus emblica beta-glucan binding protein-silver nanoparticles, *SIV* Subintestinal vein, *SmPNPs* Spirulina maxima-derived pectin nanoparticles, *WHP* Wound healing percentage, *WHR* Wound healing rate

* Indicates statistically significant values (*p* < 0.05) as reported in the original studies

Among silver nanoparticle (AgNP) interventions, accelerated wound closure was observed across the evaluated models. Two studies reported closure rates over 90% within the first two weeks of treatment [49, 51]. Additionally, one study demonstrated that the delivery route affected healing efficacy, as AgNPs administration via water immersion yielded faster healing and higher closure percentages than direct topical application [14].

Chitosan-based nanoparticle formulations also accelerated wound closure. Peptide-loaded formulations, such as Octominin-CNPs, achieved a wound healing percentage over 91% by day 21, which was the highest healing rate compared to vehicle controls [15]. For zinc oxide formulations, *β*-chitosan-derived ZnO nanoparticles increased wound closure rates compared to both chitosan alone and PBS controls across all time points up to 15 days [45]. Additionally, a chitosan-silver nanocomposite cleared female parasites within 48 h and completed wound healing within 7 days, preventing reinfection during the two-week monitoring period [50].

Other nano-delivery systems enhanced wound healing through different mechanisms and administration routes. A dose-dependent acceleration in macroscopic healing was reported by Tayyeb et al. [46], where higher nanoparticle concentrations led to better wound closure rates by the second week compared to lower doses. Electrospun scaffolds, such as curcumin-berberine-loaded PCL nanofibers, increased healing rates compared to individual components and control groups [43], while topical ethosome nanoemulsions led to near-complete closure by the third week [48].

For alternative delivery routes, oral administration of resveratrol-loaded liposomes accelerated healing timelines compared to untreated models [47]. Additionally, selenium-based nanomaterials accelerated skin wound closure and increased caudal fin regeneration rates in adult zebrafish [42].

In addition to wound size reduction, qualitative tissue improvements were observed macroscopically. Four studies reported the restoration of skin pigmentation at the wound margins [15, 44, 48, 49]. Nanomaterial treatments also resulted in earlier blood vessel formation and complete re-epithelialization with minimal or no visible scar tissue.

#### Microscopic wound healing

Microscopic evaluation was conducted in 9 out of the 12 studies to assess tissue structure (Table 2). Eight studies used histopathological analysis with H&E staining. In contrast, one study used fluorescence microscopy in transgenic models to observe real-time cellular and vascular responses.

##### Inflammation modulation and angiogenesis (6 h to 7 days post-wounding)

During the early phases of healing, nanomaterial treatments accelerated immune cell recruitment and limited prolonged inflammation. At 6 h post-injury, Nano-Se@S increased the infiltration of neutrophils and macrophages to the fin wound site [42]. Similarly, AgNP administration resulted in immune cell aggregation at the wound margins [14]. Untreated controls showed persistent inflammation and necrosis during the first week. In contrast, nano-treated groups showed leukocyte infiltration followed by granulation tissue formation and early collagen deposition between days 2 and 5 [44, 51]. By day 7, treatments such as Octominin-CNPs and SmPNPs resulted in reduced tissue necrosis and lower inflammation levels [15, 44].

Nanomaterials also induced neovascularization at the injury site. Fluorescence imaging showed that Nano-Se@S stimulated angiogenesis by increasing subintestinal vein (SIV) sprouting within 48 h, while preventing vessel narrowing caused by oxidative stress [42]. Additionally, chitosan-silver nanocomposites induced the early formation of healthy blood capillaries within the treated zones [50].

##### Structural tissue regeneration and re-epithelialization (5 to 16 days post-wounding)

Nanoparticle-treated groups showed organized tissue maturation following the early vascular response. Between days 5 and 7, nanomaterials promoted the filling of the wound cavity, forming a thick epithelial layer, dense dermal architecture, and early skeletal muscle regeneration [14, 15]. For instance, SmPNPs-treated models developed a dense neoepidermis measuring 53 μm in thickness at day 7, compared to 18 μm in vehicle controls [44].

During the later phases (Days 10 to 16), these processes resulted in complete tissue repair. At day 10, control groups lacked epithelialization, while AgNP-treated wounds achieved full recovery of the epithelial architecture with mature, well-organized collagen [51]. By days 14 to 16, delivery systems including zinc oxide-chitosan nanoparticles [46] and curcumin-berberine nanofibers [43] resulted in full re-epithelialization, dermal restoration, and skeletal muscle regeneration with minimal localized inflammation, leading to the replacement of the wound defect with newly formed skin and scales [49].

### Gene expression and antioxidant outcomes

Eight out of 12 studies included molecular evaluations (Table 3). Seven studies used quantitative real-time PCR (qRT-PCR) for gene expression [14, 15, 43–46, 48], and one study used global RNA-sequencing (RNA-Seq) for transcriptomic profiling [42]. The evaluated molecular targets were categorized into inflammatory cytokines, growth factors, antioxidant enzymes, and extracellular matrix (ECM) remodeling markers.

**Table 3:** Gene expression and antioxidant outcomes

Author	Evaluation Method	Main Outcomes
Dias et al. [15]	Molecular (qPCR) profile	At 2 dpw: ↑ *IL1Β* (11.94 ± 0.42-fold)^*^, ↑ *TNFΑ*, ↑ *IL10*, ↑ *cxcl18b*, *↑ ccl34a4*, ↑ *MMP9* versus vehicle. At 7 dpw: ↓ *TNFΑ*, *↓ IL1Β*, ↓ *cxcl18b*, *↓ ccl34a4*, ↓ *MMP9* versus Octominin group.
Seo et al. [14]	Molecular (qPCR) profile	At 12 hpw: *↑ IL-1β* (Wounded: 14.0-fold, Direct: 10.5-fold, Immersion: 5.9-fold)^*^, ↑ *MMP-9* (Wounded: 182.6-fold, Direct: 97.5-fold, Immersion: 73.7-fold)^*^, ↑ *MMP-13* (Wounded: 3.4-fold, Direct: 3.9-fold, Immersion: 2.9-fold)^*^ (showing reduced elevation in nano-treated groups). Also, ↓ *TNF-α* (0.3-0.4-fold)^*^, ↓ *TGF-β* (Direct: 0.5-fold, Immersion: 0.2-fold)^*^ were down-regulated across all wounded At 24 hpw: ↑*TGFβ* (1.6-fold in immersion only; ↓ in other groups)^*^, ↑*TNF-α* (Wounded: 1.8-fold, Direct: 2.5-fold, Immersion: 2.0-fold)^*^, ↓*MMP9*, *MMP13*, and *IL-1β* (sharply decreased toward baseline in nano-treated groups compared to wounded control). At 5 dpw: Inflammatory and remodeling markers back to near-basal levels (except slight induction remaining for *MMP9 and IL1β*. Antioxidant gene: At 12 hpw: ↑SOD (Immersion: 2.0-fold, Direct: 1.2-fold)^*^ vs. ↓wounded control (0.8-fold). At 24 hpw: ↑CAT in direct group (6.3-fold)^*^ vs. immersion group returned to baseline.
Iswarya et al. [49]	Molecular (qPCR) profile	Not Evaluated.
Tayyeb et al. [46]	Molecular (qPCR) profile	At 80 µg/mL dose: Inflammatory Cytokines: *↓ TNF-Α* (3.4-fold vs. Control: 5.7-fold)^*^ and *↓ IL-1Β* (2.3-fold vs. Control: 4.6-fold)^*^. Matrix Remodeling: *↓ MMP13* (1.7-fold vs. Control: 3.5-fold)^*^, preventing extracellular matrix degradation
Zain et al. [48]	Molecular (qPCR) profile	At 2 dpw: *TGF-β*: Showed less suppression (↓0.18- to 0.28-fold)^*^ compared to untreated control ↓0.05-fold). *IL1β*: Upregulated by OPL-FEE-NE (↑2.8-fold)^*^ and OPL-FEE (↑2.1-fold)^*^, remaining lower than untreated control (↑6.9-fold). *TNFα*: Suppressed in treated groups ↓0.18- to 0.21-fold)^*^ compared to untreated control (↑5.2-fold). Matrix Markers (*MMP9*,* MMP13*,* TIMP2B*): Slight upregulation in OPL-FEE and OPL-FEE-NE (mmp9 at ↑8.7- to 9.0-fold)^*^, significantly lower than untreated control peaks (mmp9 at ↑26.8-fold)^*^. Antioxidant gene: SOD1: Highly upregulated in all treated groups (↑3.9- to 4.7-fold)^*^ compared to untreated control ↑3.0-fold). CAT: Upregulated in all treated groups, reaching ↑3.4-fold^*^ for OPL-FEE and ↑2.4-fold^*^ for OPL-FEE-NE. At 8 dpw : *TGFβ*: Further downregulated in all groups, with OPL-FEE-NE showing stronger suppression (↓0.03- to 0.09-fold)^*^ than OPL-FEE. Inflammatory Cytokines (*IL1β*,* TNFα*): Systemic suppression in all treated groups (IL1β at ↓0.06- to 0.08-fold^*^; *TNFα* at ↓ 0.02- to 0.03-fold)^*^. Matrix Markers: Consistently downregulated in all treated groups: *MMP9* (↓0.063- to 0.068-fold)^*^, *MMP13* ↓ 0.08- to 0.44-fold)^*^, and *TIMP2B* (↓ 0.03- to 0.08-fold)^*^. Antioxidant gene: (SOD1, CAT): Expressions decreased back to healthy levels, similar to the untreated control group.
Ramachandran et al. [45]	Molecular (qPCR) profile	(Observed over 15 days): (↓*IL-6 & ↓ TNF-α*): β-Ch-ZnO-NPs group showed the strongest decrease compared to β-Ch alone, while the Control group remained high. (↑*IL-2*): Highest increase observed in β-Ch-ZnO-NPs group compared to β-Ch alone and Control. ↑*Bax & ↓Bcl*-2): β-Ch-ZnO-NPs group regulated both genes significantly better than β-Ch alone, while Control showed no change
Kandaswamy et al. [43]	Molecular (qPCR) profile	At 14 dpw: Cu@Bbc group significantly suppressed markers compared to controls by downregulating ↓ IL-1βto 1.3-fold^*^ (vs. 3.3-fold), ↓ TNFα to 2.3-fold^*^ (vs. 6.0-fold), and ↓ MMP13 to 1.4-fold^*^ (vs. 4.5-fold).
Yu et al. [47]	Molecular (qPCR) profile	Not Evaluated.
Rajapaksha et al. [44]	Molecular (qPCR) profile	By 24 dpw: In the wounded tissue, SmPNPs enhanced the healing cascade compared to the vehicle group by upregulating early inflammatory genes (↑*TNFα* at 1 dpw: 18.74-fold^*^ vs. 6.50-fold; ↑*IL1β* at 7 dpw: 5.75-fold^*^), chemokines (↑*cxcl18b*,* ↑ccl34a.4*), tissue remodeling factors (↑*TGFβ1* at 2 dpw: 12.71-fold^*^ vs. 4.32-fold; ↑*mmp9* at 2 dpw: 10.01-fold^*^; ↑*mmp13*), and maintaining a prolonged anti-inflammatory response via *il10* (> 2-fold)^*^ up to 24 dpw. Antioxidant gene: At 2 dpw: SmPNPs increased antioxidant expression at the wound site (↑CAT to 23.45-fold^*^ and SOD1 to 14.92-fold^*^.
Abu-Elala et al. [50]	Molecular (qPCR) profile	Not Evaluated.
Ravindran et al. [51]	Molecular (qPCR) profile	Not Evaluated.
Cao et al. [42]	Transcriptomics (RNA-Seq).	During healing: Global RNA-Seq of the regenerating zebrafish fin showed that Nano-Se@S upregulated 218 genes and downregulated 295 genes, specifically activating the FGF signaling pathway (via *fgf7*) and the Jak-STAT pathway (via *il20ra* and *il13ra1*), while promoting biomolecular synthesis and redox processes.
	Biochemical (DHE assay)	Dose-dependent ROS scavenging significantly reduced H2O2-induced vascular damage (narrowing and malformations) in a transgenic zebrafish model.

*Bax* Bcl-2 associated X protein, *Bcl-2* B-cell lymphoma 2, *CAT* Catalase, *ccl34a4* Chemokine (C-C motif) ligand 34a4, *Cu@Bbc* Curcumin-berberine chloride, *cxcl18b* Chemokine (C-X-C motif) ligand 18b, *DHE* Dihydroethidium, *dpw* Days post-wounding, *FGF* Fibroblast growth factor, *H₂O₂* Hydrogen peroxide, *hpw* Hours post-wounding, *IL* Interleukin, *Jak-STAT* Janus kinase/signal transducers and activators of transcription, *MMP* Matrix metalloproteinase, *Nano-Se@S* Selenium-sulfur core-shell nanoparticles, *OPL-FEE* 1-Octyl-D-Phenylalanine-functionalized *Euphorbia hirta* ethanolic extract, *OPL-FEE-NE* 1-Octyl-D-Phenylalanine-functionalized *Euphorbia hirta* ethanolic extract nanoemulsion, *qPCR* Quantitative polymerase chain reaction, *RNA-Seq* RNA sequencing, *ROS* Reactive oxygen species, *SmPNPs* *Spirulina maxima*-derived pectin nanoparticles, *SOD* Superoxide dismutase, *TGF-β* Transforming growth factor beta 1, *TIMP2b* Tissue inhibitor of metalloproteinase 2b, *TNF-α* Tumor necrosis factor alpha; *ZnO-NPs* Zinc oxide nanoparticles

* Indicates statistically significant values (*p* < 0.05) as reported in the original studies

Inflammatory markers were evaluated in 7 of the 8 molecular studies. Across 6 studies, nanomaterial treatments resulted in the downregulation of pro-inflammatory markers, specifically *TNF-α*,* IL-1β*, and *IL-6* back to near-basal levels between days 5 and 15 post-wounding, whereas untreated controls maintained elevated expression levels [14, 15, 43, 45, 46, 48]. Conversely, nanoparticle treatments sustained or upregulated the expression of the anti-inflammatory cytokine *IL-10* [15, 44], with SmPNPs maintaining an *IL-10* upregulation of over 2-fold up to day 24 post-wounding [44]. Additionally, one study reported an upregulation of *IL-2* expression in treated groups compared to controls [45]. For chemokine regulation, Octominin-CNPs [15] upregulated the chemokines cxcl18b and ccl34a4 at day 2, followed by their downregulation by day 7. Similarly, SmPNPs [44] induced the upregulation of both cxcl18b and ccl34a4 at the wound site.

The growth factor *TGF-β* was evaluated in 3 studies and varied based on the specific nanomaterial and time point. At day 2 post-wounding, SmPNPs upregulated TGF-β expression by 12.71-fold [44], while the nanoencapsulated formulation OPL-FEE-NE showed less suppression of this growth factor compared to both the raw formulation and untreated controls [48]. By day 8, TGF-β expression decreased across all treatments, with OPL-FEE-NE maintaining lower expression levels than raw OPL-FEE [48]. Additionally, the administration route of silver nanoparticles (AgNPs) altered expression patterns at 24 h; water immersion resulted in a 1.6-fold upregulation of *TGF-β*, whereas direct topical application caused its downregulation [14].

Antioxidant gene expression, specifically superoxide dismutase (SOD) and catalase (CAT), was evaluated in 3 studies. Within the first 12 to 48 h post-wounding, nanoparticle interventions resulted in the upregulation of these genes. Specifically, silver nanoparticles (AgNPs) via water immersion increased SOD expression by 2.0-fold at 12 h [14], OPL-FEE-NE upregulated both SOD and CAT expression [48], and SmPNPs increased CAT and SOD1 expression by 23.45-fold and 14.92-fold, respectively, at day 2 [44]. By day 8, these expression levels returned to baseline [48]. Additionally, apoptosis regulation was evaluated in one study, where zinc oxide nanoparticles (ZnO-NPs) downregulated the pro-apoptotic gene Bax and upregulated the anti-apoptotic marker Bcl-2 over 15 days [45].

Markers associated with matrix remodeling, particularly matrix metalloproteinases (MMPs) and tissue inhibitors of metalloproteinases (TIMPs), were evaluated in 6 studies. Nanomaterial treatments generally led to a downregulation of remodeling enzymes in the later healing stages. Specifically, OPL-FEE-NE downregulated both *MMP9* and *MMP13* by day 8 [48]. In a single study evaluating inhibitor expression, this same treatment downregulated *TIMP2b* at day 8 [48]. Cu@Bbc decreased *MMP13* expression at day 14 [43], and a high dose of nanomaterials reduced mmp13 [46]. Under Octominin-CNPs treatment, mmp9 expression was downregulated by day 7 post-wounding [15].

However, early expression patterns varied across these delivery systems. At day 2, SmPNPs upregulated both *MMP*9 and *MMP*13 expression before progressing toward later stages [43]. Furthermore, the administration route altered enzyme levels at 12 h post-wounding, as silver nanoparticle (AgNP) immersion limited the elevation of *MMP9* and *MMP13* more effectively than direct topical application, and both routes returned these remodeling markers back toward near-basal levels by day 5 [14].

Cao et al. [42] identified 218 upregulated and 295 downregulated genes using in vivo RNA-Seq on the regenerating zebrafish fin. Gene Set Enrichment Analysis (GSEA) revealed decreased immune system processes, increased redox networks, and upregulation of the FGF signaling pathway (via fgf7) and Jak-STAT pathway (via il20ra and il13ra1).

### Risk of bias assessment

**Fig. 4:**
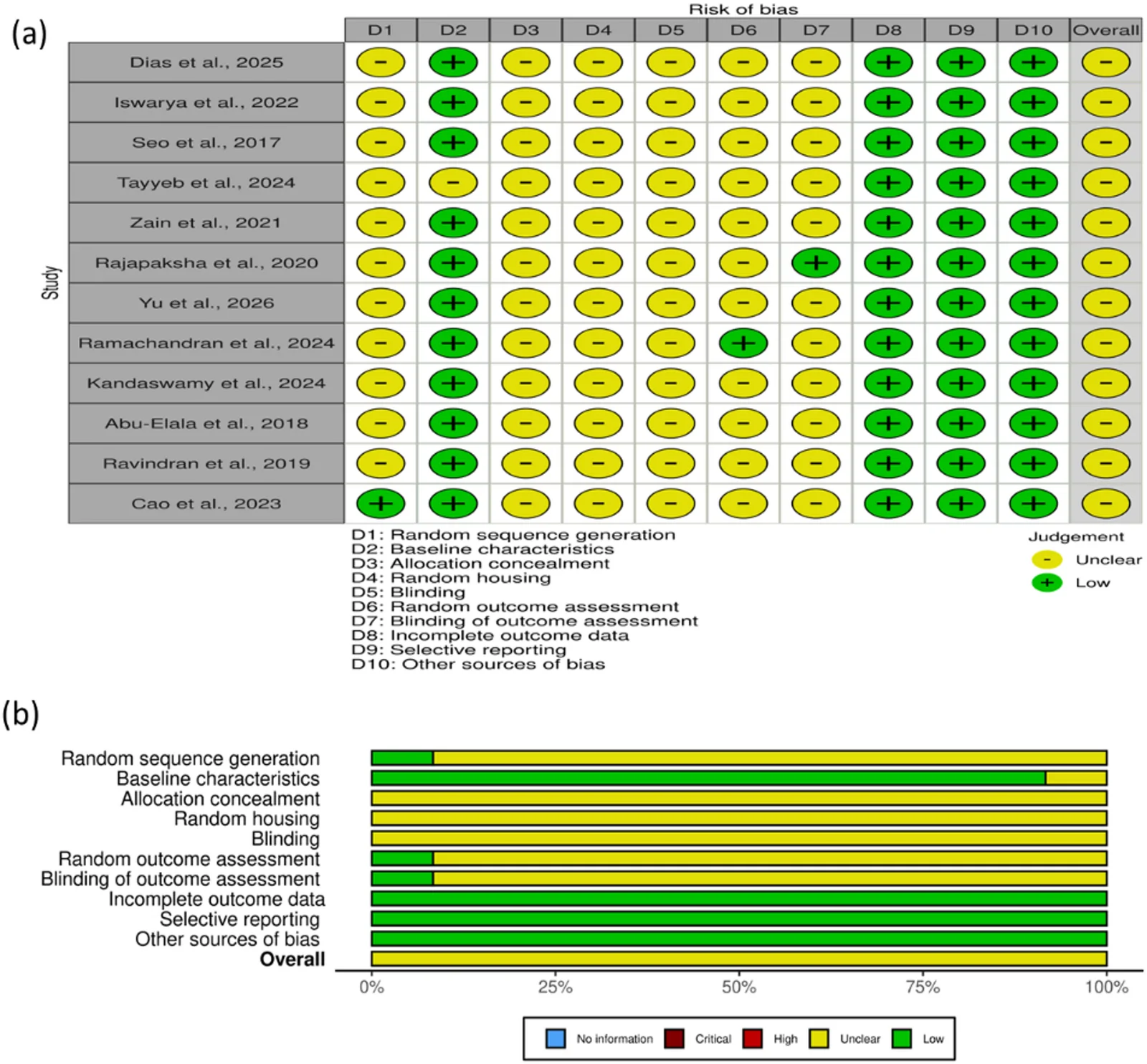
Risk of bias in the 12 in vivo studies on nanomaterials for fish wound healing, using the SYRCLE tool. **a** Traffic light plot showing the risk for each study. **b** Summary bar plot showing overall risk percentage

The methodological quality of the 12 included studies was evaluated using the SYRCLE risk of bias tool. The specific judgments for each domain are presented in the traffic light plot (Fig. 4a), while the overall distribution of low and unclear risk across the studies is shown in the summary bar plot (Fig. 4b).

The evaluation showed a low risk of bias for reporting and attrition domains, but an unclear risk for performance and detection domains due to insufficient reporting. Across the SYRCLE domains, incomplete outcome data (Domain 8), selective outcome reporting (Domain 9), and other sources of bias (Domain 10) were rated as low risk in all 12 studies (100%). These papers accounted for all animals, matched their methods with outcomes, and included ethical approvals and conflict of interest statements. Baseline characteristics (Domain 2) carried a low risk of bias in 11 out of 12 studies (91.7%) because they used standardized, age-, and weight-matched fish across groups, whereas only Tayyeb et al. [46] was rated as unclear due to a lack of baseline descriptive data.

Conversely, allocation concealment (Domain 3), random housing (Domain 4), and investigator blinding (Domain 5) were rated as unclear risk in all studies, as none of the authors detailed how they concealed group allocation or randomized tank distribution. Random outcome assessment (Domain 6) and blinding of outcome assessors (Domain 7) were also predominantly marked as unclear risk. The only exceptions were Ramachandran et al. [45], which reported a low risk for random outcome assessment, and Rajapaksha et al. [44], which reported a low risk for blinding of outcome assessors. For random sequence generation (Domain 1), 11 studies were evaluated as unclear, leaving Cao et al. [42] as the only paper to explicitly describe its methodology for generating a random allocation sequence.

## Discussion

### Overview of findings

This systematic review analyzes twelve studies published between 2017 and 2026 on nanoparticle-based biomaterials for skin wound healing in in vivo teleost models. The primary finding is that nanoparticle treatments accelerated wound healing compared to untreated controls, and in some cases, yielded better results than standard therapies. Treated groups showed faster wound closure, enhanced re-epithelialization, improved granulation tissue formation, reduced inflammation, and improved expression in molecular repair markers [14, 15, 42–44, 46]. It is important to note that the mechanistic pathways discussed herein are inferred across disparate, single-study findings rather than established through direct comparative trials or causal validation.

The reviewed studies show a clear shift in the types of materials used over time. Early research focused on metallic nanoparticles, mainly AgNPs and ZnO NPs. These materials provided strong antimicrobial effects and controlled reactive oxygen species (ROS) to prevent infection and support tissue repair [14, 45, 49, 51]. However, recent studies (2025–2026) are moving away from metals toward biopolymer-based nanoparticles. This change is due to concerns about the toxicity of metals, including their accumulation in the body, oxidative stress at high doses, and environmental risks [52, 53]. In contrast, biopolymers especially chitosan and liposomes are biodegradable, highly biocompatible, and can easily deliver various active agents [54, 55].

Macroscopic analysis showed high wound closure rates across the different materials. Silver nanoparticles achieved over 90% healing within two weeks in several fish models [49, 51], while biopolymeric systems like Octominin-loaded chitosan nanoparticles reached a 91.73% closure rate by day 21 [15]. Microscopic evaluation suggested significant tissue repair. For instance, Spirulina-derived pectin nanoparticles produced a 53 μm thick neoepidermis by day 7, compared to just 18 μm in controls [44]. The delivery method also influenced these outcomes. Water immersion generally performed better than direct topical application, likely because it keeps the nanoparticles in continuous contact with the wound while minimizing handling stress [14].

### Mechanisms of nanoparticle-mediated tissue regeneration

The molecular and cellular processes through which nanomaterials promote fish wound healing rely on four connected actions, including antimicrobial activity, immunomodulation and accelerated inflammatory resolution, antioxidant and ROS-scavenging activity, and the stimulation of angiogenesis and extracellular matrix (ECM) remodeling, which work together over time to create a favorable environment for rapid, scar-free tissue repair.

First, nanomaterials provide potent antimicrobial and anti-biofilm activity. A key advantage of nanoparticle treatments is their potent bactericidal activity and biofilm clearance, which prevents secondary infections and the severe inflammation that often hinders the healing process in water. Metallic nanoparticles, especially AgNPs and ZnO NPs, kill a broad range of common fish pathogens such as *Aeromonas hydrophila*, *S. aureus*, and *P. aeruginosa* by damaging bacterial walls, generating ROS, and releasing metal ions that block vital bacterial enzymes [45, 46, 49]. For instance, ZnO-CN NPs showed strong antibacterial activity at a concentration of 40 µg/mL [46]. Beyond metals, organic systems like curcumin-berberine chloride nanofibers reduced methicillin-resistant *S. aureus* growth by 87% and damaged mature bacterial biofilms by 81% [43], establishing a reduced microbial burden for tissue repair. Similarly, in parasite-infected models, a chitosan-silver nanocomposite cleared female *Lernaea cyprinacea* parasites within 48 h. This dual action led to full wound healing and prevented reinfection [50]. These findings suggest that pathogen control is an important step for nanomaterials to accelerate healing, especially in aquaculture where opportunistic infections are common.

Second, they promote immunomodulation and accelerated inflammatory resolution. Another key way nanomaterials facilitate healing is by controlling the timing of inflammation. Healing starts with a rapid rise in inflammatory signals (cytokines and chemokines) to clear cellular debris, which must drop later to prevent harmful, chronic inflammation. For example, Octominin-loaded chitosan nanoparticles increased early inflammatory signals (*IL-1β*, *TNF-α*, *cxcl18b*, and *ccl34a4*) at day 2 to recruit immune cells, but these levels dropped significantly by day 7 [15]. Also, Spirulina-derived pectin nanoparticles raised *TNF-α*, *IL-1β*, and *TGF-β* levels early on, while keeping the anti-inflammatory signal *IL-10* active to maintain balanced healing [44]. This balanced pattern was consistent across other nanomaterials. Silver nanoparticles stopped a harmful, sharp rise of *IL-1β* at 12 h, while increasing *TGF-β* by 24 h to promote cellular migration into the wound [14]. Similarly, zinc oxide nanoparticles lowered *TNF-α* and *IL-1β* levels [46], while a nanoemulsion of oil palm leaf extract (OPL-FEE-NE) rapidly reduced inflammatory signals as the wound transitioned to the rebuilding phase [48]. These results suggest that controlling the timing of these signals appears to be a key factor in why nanomaterials are more effective than traditional treatments, which often fail to manage the stages of inflammation.

Third, antioxidant and ROS-scavenging activity. Maintaining redox homeostasis is essential for proper healing, as excessive ROS causes cellular damage, while insufficient levels weaken the body’s defense against bacteria. When a fish is injured, a sudden increase in ROS occurs. If this is not controlled, it causes tissue necrosis, which impairs tissue regeneration. Several nanomaterials protect cells by upregulating natural antioxidant genes. For example, silver nanoparticles doubled the expression of the protective gene SOD at 12 h [14]. Also, Spirulina pectin nanoparticles increased other key antioxidant genes, *cat* and *sod1*, by over 14 to 23 times at day 2 [44]. Similarly, a selenium-sulfur nanocomposite (Nano-Se@S) neutralized these deleterious ROS to protect developing blood vessels from oxidative damage [42]. This protection preserves the surrounding muscle architecture and blood vessels. For instance, a nanoemulsion made from oil palm leaf extract (OPL-FEE-NE) increased *sod* and *cat* levels right after wounding, and these levels returned to normal by day 8 as the tissue recovered [48]. Zinc oxide nanoparticles also directly protected cellular viability by regulating apoptosis. Over 15 days, they downregulated *Bax* (a pro-apoptotic gene) and upregulated *Bcl-2* (an anti-apoptotic gene) to prevent cell death [45]. These results suggest that removing ROS and preventing cellular apoptosis appears to be an essential process through which nanomaterials create a healthy environment for clean tissue repair.

Fourth, stimulation of angiogenesis and ECM remodeling. These processes are essential for delivering oxygen and nutrients to the wound while replacing temporary tissue with organized collagen. A selenium-sulfur nanocomposite (Nano-Se@S) enhanced angiogenesis by stimulating vein sprouting in zebrafish larvae within 48 h, while preventing vessel narrowing [42]. Similarly, chitosan-silver nanocomposites induced early capillary formation, which improved tissue perfusion and accelerated wound closure [50]. At the molecular level, nanomaterials regulate the enzymes responsible for tissue remodeling. Matrix metalloproteinases (MMPs), such as *MMP9* and *MMP13*, are needed for initial matrix degradation, but must be downregulated later to prevent excessive collagen breakdown. Nanomaterials maintain this timing: Spirulina-derived pectin nanoparticles upregulated *mmp9* and *mmp13* at day 2 to help clear the initial matrix [44], while an oil palm leaf extract nanoemulsion (OPL-FEE-NE) downregulated *MMP9*, *MMP13*, and *TIMP2b* by day 8 to allow organized collagen deposition [48]. Additionally, Nano-Se@S upregulated key signaling pathways (*FGF7*, *il20ra*, and *il13ra1*), consistent with growth-factor-mediated tissue repair [42]. These findings suggest that nanomaterials promote functional tissue regeneration primarily by coordinating angiogenesis and balancing ECM remodeling.

These four mechanisms work together to optimize healing. Antimicrobial activity prevents infection; immunomodulation ensures a timely inflammatory response and its resolution; antioxidant protection preserves cellular viability; and angiogenesis and ECM remodeling facilitate adequate blood supply and structural repair. This coordinated action makes nanomaterials more effective than conventional treatments for wound healing in aquaculture.

### Limitations of the current literature

Despite promising findings, the practical application of nanomaterials in commercial aquaculture is limited by several critical gaps. First, the validity of the current evidence is constrained by methodological gaps in the literature. The SYRCLE assessment showed a widespread lack of researcher blinding, random animal housing, and allocation concealment across the studies. These reporting deficiencies make it difficult to exclude systematic bias, meaning the reported healing efficacy of these nanomaterials might be overestimated due to potential publication bias. Second, current research shows a strong species bias, as 75% of studies focused on laboratory-reared zebrafish (*Danio rerio*). Although zebrafish serve as practical models due to their genetics and tissue transparency [56], translating these laboratory findings to large, commercially farmed species presents practical challenges. Notably, only one study evaluated Mozambique tilapia (*Oreochromis mossambicus*) [49], and no studies evaluated Nile tilapia (*Oreochromis niloticus*), one of the most economically important aquaculture species globally [57]. Addressing this gap is essential to successfully transfer nanomaterial treatments from controlled laboratory settings to practical fish farming. Third, substantial variations in nanoparticle types, dosages, and delivery routes across studies prevented a quantitative meta-analysis and limited direct comparisons between them. Fourth, the long-term safety of these treatments remains poorly characterized. Most studies monitored healing for fewer than 30 days without tracking the long-term accumulation of metallic nanoparticles (such as silver and zinc oxide) in fish tissues. Fifth, assessment methods were predominantly basic and invasive, relying on terminal sampling and standard H&E staining. No study applied non-invasive monitoring tools like ultrasound. Furthermore, advanced techniques such as Masson’s trichrome staining for collagen, IHC, and SEM were rarely used, limiting detailed insights into tissue recovery. Finally, research on advanced hybrid biopolymer systems specifically combining nano-hyaluronic acid and nano-chitosan has not yet been explored in aquaculture. The lack of studies testing this hybrid biomaterial in fish models, particularly Nile tilapia, represents a major gap in the literature.

## Conclusion and future directions

This systematic review shows that nanomaterials significantly accelerate wound healing in teleost fish by promoting tissue regeneration and modulating key molecular pathways. The gradual shift from metallic nanoparticles to biopolymer-based systems reflects a growing focus on biocompatibility and safety in aquaculture. However, applying these laboratory findings to practical farming requires addressing key gaps, such as the heavy bias toward zebrafish, methodological limitations, and the lack of long-term safety data in fish tissues. Notably, testing hybrid biopolymer systems specifically combining nano-hyaluronic acid and nano-chitosan remains completely unexplored in fish models, particularly the Nile tilapia (*Oreochromis niloticus*). This gap is crucial to address due to the complementary biological properties of these materials and the high global economic value of the Nile tilapia. Evaluating this hybrid biomaterial under real farming conditions will help improve skin healing, support the immune system, and provide a safe and effective wound treatment for sustainable aquaculture.

## Supplementary Information

Supplementary Material 1.

## Data Availability

All data generated or analyzed during this study are included in this published article.

## Abbreviations

AgNPs: Silver nanoparticles
CAT: Catalase
Cu@Bbc: Curcumin-berberine chloride
DHE: Dihydroethidium
ECM: Extracellular matrix
FGF: Fibroblast growth factor
GSEA: Gene Set Enrichment Analysis
H&E: Hematoxylin and Eosin
IHC: Immunohistochemistry
IL: Interleukin
Jak-STAT: Janus kinase/signal transducers and activators of transcription
MeSH: Medical Subject Headings
MMPs: Matrix metalloproteinases
Nano-Se@S: Selenium-sulfur nanocomposite
Octominin-CNPs: Octominin-loaded chitosan nanoparticles
OPL-FEE-NE: Oil palm leaf extract nanoemulsion
OSF: Open Science Framework
PBS: Phosphate-buffered saline
PCL: Polycaprolactone
PICOS: Population, Intervention, Comparison, Outcome, Study design
PRISMA: Preferred Reporting Items for Systematic Reviews and Meta-Analyses
qRT-PCR: Quantitative real-time polymerase chain reaction
RNA-Seq: RNA sequencing
ROS: Reactive oxygen species
SEM: Scanning electron microscopy
SIV: Subintestinal vein
SmPNPs: Spirulina-derived pectin nanoparticles
SOD: Superoxide dismutase
SWiM: Synthesis Without Meta-analysis
SYRCLE: Systematic Review Centre for Laboratory Animal Experimentation
TGF-β: Transforming growth factor-beta
TIMPs: Tissue inhibitors of metalloproteinases
TNF-α: Tumor necrosis factor-alpha
WHP: Wound healing percentage
WHR: Wound healing rate
ZnO NPs: Zinc oxide nanoparticles
ZnO-CN NPs: Zinc oxide-chitosan nanoparticles
